# Slow‐Oscillatory Transcranial Alternating Current Stimulation Over the Primary Motor Cortex Improves Motor Skill Acquisition

**DOI:** 10.1111/ejn.70269

**Published:** 2025-09-26

**Authors:** Shota Miyaguchi, Yasuto Inukai, Kanta Igarashi, Shunpei Yamamoto, Naofumi Otsuru, Hideaki Onishi

**Affiliations:** ^1^ Institute for Human Movement and Medical Sciences Niigata University of Health and Welfare Niigata Japan; ^2^ Graduate School Niigata University of Health and Welfare Niigata Japan

**Keywords:** motor learning, primary motor cortex, slow oscillation, transcranial alternating current stimulation, visuomotor tracking task

## Abstract

Slow‐oscillatory brain activity (< 4 Hz) is crucial in memory consolidation and motor performance. Although transcranial alternating current stimulation (tACS) can modulate cortical oscillatory activity and influence motor learning, the effects of slow oscillation modulation via tACS remain unclear. This study investigated the effects of 0.75‐Hz tACS applied to the primary motor cortex (M1) on the acquisition and retention of a visuomotor tracking task. Fifty‐four right‐handed healthy adults (mean age: 21.2 ± 0.6 years) were assigned to one of three groups: M1‐tACS, Cz‐tACS, or sham. In the M1‐tACS and sham groups, electrodes were placed over the right M1 and left supraorbital ridge. In the Cz‐tACS group, electrodes were placed over the Cz (International 10–20 system) and left supraorbital ridge to assess site specificity. tACS was administered for 30 min at 0.75 Hz and 1.0 mA, while participants practiced the visuomotor task. A retention test was conducted the following day. Motor learning was assessed using the power approximation index from the learning curve and error rates. The M1‐tACS group showed a significantly lower approximation index than the sham group (*p* = 0.033), indicating enhanced learning. Error rates immediately after practice and on the following day were also significantly lower in the M1‐tACS group (*p* = 0.039 and *p* = 0.007, respectively). No significant differences were found in the Cz‐tACS group. These results suggest that slow‐oscillatory tACS targeting the M1 facilitates motor skill acquisition and retention, with effects specific to the hand area of M1.

## Graphical abstract

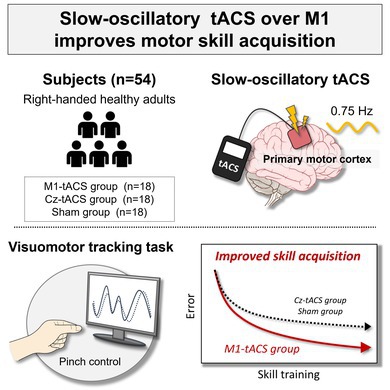

**Abbreviations**
Cz: central midline
EEG: electroencephalography
IQR: interquartile range
M1: primary motor cortex
tACS: transcranial alternating current stimulation
TMS: transcranial magnetic stimulation

## 1 Introduction

Slow‐oscillatory brain activity in the cerebral cortex is primarily observed during sleep, where it plays a crucial role in sleep‐dependent neural plasticity and memory consolidation (Giovanni et al. 2017; King et al. 2017). In the motor cortex, such low‐frequency rhythms have also been linked to movement direction and speed control during motor execution (Bansal et al. 2011; Mollazadeh et al. 2011). Moreover, these oscillations are implicated in motor error processing, with reduced network activity in the sensorimotor cortex following motor errors (Yordanova et al. 2024). Evidence further suggests a role in poststroke recovery, as increased low‐frequency power correlates with improved motor function in stroke model rats (Ramanathan et al. 2018). Together, slow‐oscillatory dynamics appear to contribute to neural plasticity and memory consolidation as well as to motor execution and error processing. Therefore, artificially modulating these brain rhythms may enhance motor learning and provide a promising avenue for therapeutic intervention.

Transcranial alternating current stimulation (tACS) is a noninvasive brain stimulation technique that modulates rhythmic neural activity in humans by delivering alternating current through scalp electrodes (Helfrich et al. 2014; Antal and Herrmann 2016). Although its effects have been extensively investigated in the alpha, beta, and gamma frequency bands (Rostami et al. 2023; McNally et al. 2024), little is known about stimulation in the slow‐wave range. For instance, applying 0.75‐Hz tACS to the frontal cortex during sleep enhanced slow‐wave activity, facilitated information encoding, and improved declarative memory retention (Marshall et al. 2006). Comparable benefits were also observed in awake participants, where stimulation during a language memory task strengthened declarative memory (Kirov et al. 2009). More recent studies have targeted the primary motor cortex (M1) in awake individuals to examine the motor effects of slow‐oscillatory tACS (Geffen et al. 2021; Bradley et al. 2022; Sale and Kuzovina 2022). Stimulation of M1 increased cortical excitability during and after the intervention (Geffen et al. 2021), and concurrent delivery to M1 and the contralateral frontal cortex during thumb abduction training facilitated next‐day retention of performance gains (Sale and Kuzovina 2022). These findings suggest that slow‐oscillatory tACS promotes motor memory consolidation. However, the site specificity of these effects remains unresolved, leaving it unclear whether they are driven by modulation of the frontal cortex or of M1. Furthermore, it is still unknown whether such stimulation can enhance skill acquisition in force control tasks that depend on sensory and error feedback, such as visual tracking, beyond its established effects on ballistic movements like thumb abduction.

As noted above, although slow‐oscillatory tACS has attracted growing interest, its effects on motor learning remain poorly understood. Therefore, this study investigated whether stimulation of the primary motor cortex (M1) with 0.75‐Hz tACS could facilitate motor skill acquisition. Participants practiced a visual tracking task while receiving M1‐targeted stimulation, and task performance was reassessed the following day to evaluate retention. To test site specificity, a control group received frontal cortex stimulation without M1 involvement. We hypothesized that tACS over M1 during motor practice would promote immediate learning and next‐day retention, whereas frontal stimulation alone would yield little or no benefit. While most previous research on motor learning with tACS has focused on alpha, beta, and gamma frequencies (Rostami et al. 2023; McNally et al. 2024), the present study aimed to extend these findings by examining the potential of slow‐wave stimulation as a novel noninvasive approach for enhancing motor learning.

## 2 Materials and Methods

### 2.1 Subjects

Fifty‐four right‐handed healthy adults (mean age: 21.2 ± 0.6 years [range: 19–22]; 28 men, 26 women) participated in this study. Handedness was assessed using the Edinburgh Handedness Questionnaire, yielding a mean laterality quotient of 94.3 ± 8.6. None of the participants reported a history of neurological, psychiatric, or orthopedic disorders requiring treatment, and all were medication‐free during the study. All participants had normal or corrected‐to‐normal vision and no contraindications to tES, such as metal implants in the head, pacemakers, seizures, or scalp or head injuries. Individuals with extensive instrumental musical or video gaming experience were excluded from recruitment. During the experimental period, participants were instructed to avoid excessive alcohol and caffeine intake and maintain adequate sleep. Participants were randomly assigned to the M1‐tACS group (*n* = 18, mean age: 21.0 ± 0.6 years, mean laterality quotient: 95.2 ± 7.2), Cz‐tACS group (*n* = 18, mean age: 21.6 ± 0.5 years, mean laterality quotient: 93.1 ± 8.5), or sham group (*n* = 18, mean age: 21.1 ± 0.3 years, mean laterality quotient: 94.6 ± 10.2). The number of participants was determined using G*Power 3.1.9.7 based on a medium effect size (f) of 0.25, an alpha level of 0.05, and a statistical power (1 − β) of 0.95. The study employed a single‐blinded design, where participants were unaware of their group assignment.

### 2.2 Visuomotor Tracking Task

**FIGURE 1:**
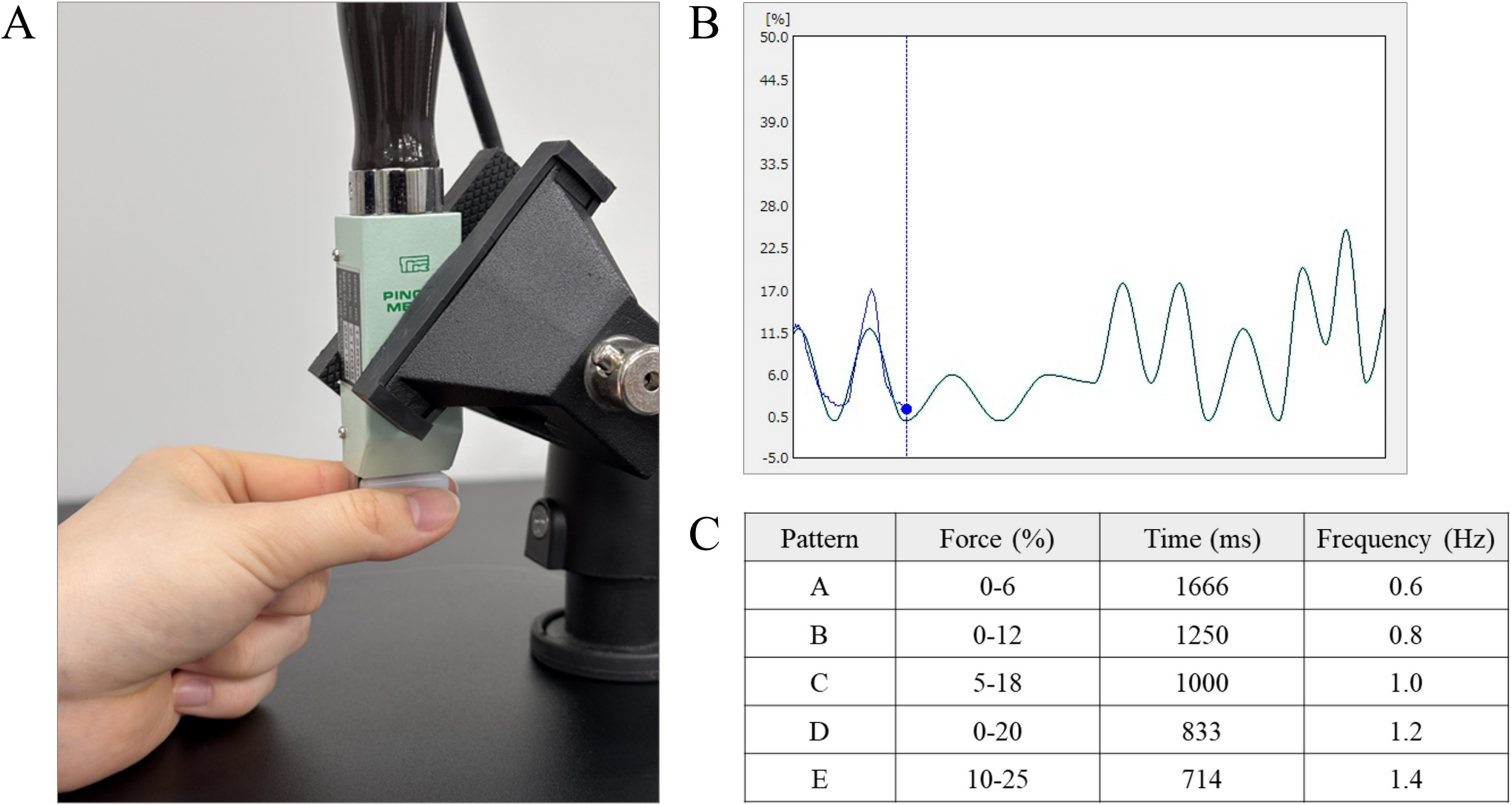
Visuomotor tracking task. (A) Pinch movement setup: Participants performed pinch movements between their left thumb and index finger, with force measurements recorded. (B) Task display: Example of target waveform (light blue curve) and target marker (blue circle) used in the task. (C) Movement patterns: five combinations of movement intensity and waveform duration used in the task.

A visuomotor tracking task—the one employed in our previous study (Yamamoto et al. 2024)—was used as the motor learning task in the present study (Figure 1). The task parameters were measured using a pinch tension meter (Takei Scientific Instruments, T.K.K. 1269n, Niigata, Japan) and force control software (Takei Scientific Instruments, Niigata, Japan). Participants sat on a chair with their left forearm resting on the armrest, operating the pinch tension meter with their left thumb and index finger (Figure 1A). The goal was to accurately align a marker, which moved up and down in response to pinch force, with a target waveform that flowed from right to left on the laptop screen (Figure 1B). Five variations of waveform times were included in the test: 714, 833, 1000, 1250, and 1666 ms. The movement intensity was set within the range of 0%–25% of the participant's maximum pinch force, with five variations (0%–6%, 0%–12%, 5%–18%, 0%–20%, and 10%–25%). This range was established to enable moderate learning without imposing an excessive burden on the participants. Five movement patterns were created by combining different movement intensities and waveform durations: pattern A (0%–6%, 1666 ms); pattern B (0%–12%, 1250 ms); pattern C (5%–18%, 1000 ms); pattern D (0%–20%, 833 ms); and pattern E (10%–25%, 714 ms) (Figure 1C). Each 60‐s trial consisted of these five movement patterns presented randomly 11 times. Participants were instructed to trace the target waveform as accurately as possible. The participants were not informed about the number of trials in the task. Instructions were uniform across all three groups. During each trial, the force control software measured the deviation in pinch tension required to match the provided target waveform at a sampling frequency of 100 Hz. The deviation data, recorded to the third decimal place, were saved in an Excel file and used for analysis. The average deviation over 60 s was calculated as the error value and normalized to each participant's maximum tension to determine the error rate for each trial.

### 2.3 Transcranial ACS

**FIGURE 2:**
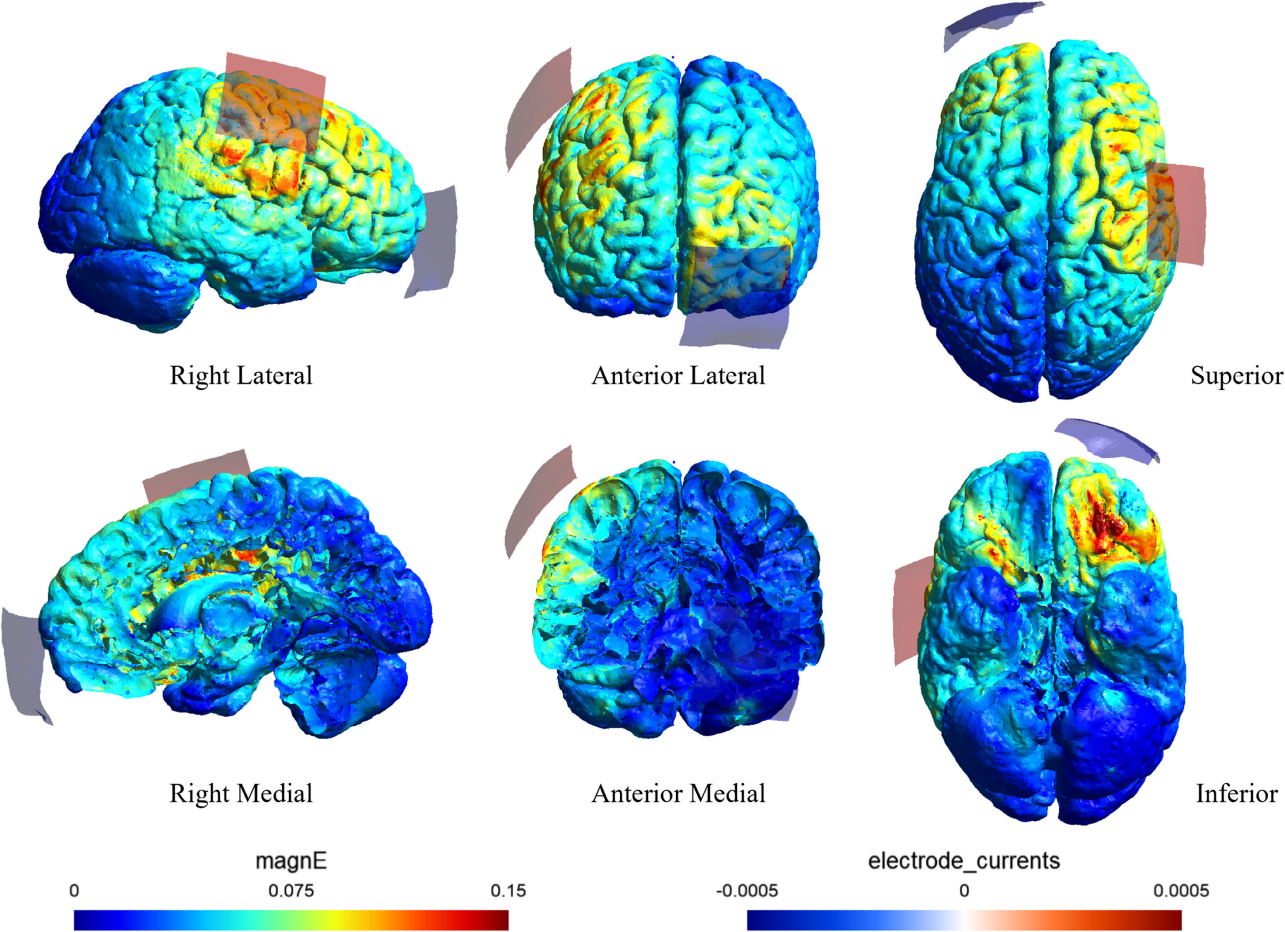
Electrode montage and electric field simulations. Electric field simulations using SimNIBS version 4.0.1 for the M1‐tACS and sham groups, illustrating the electrode montage and simulated electric field distributions.

**FIGURE 3:**
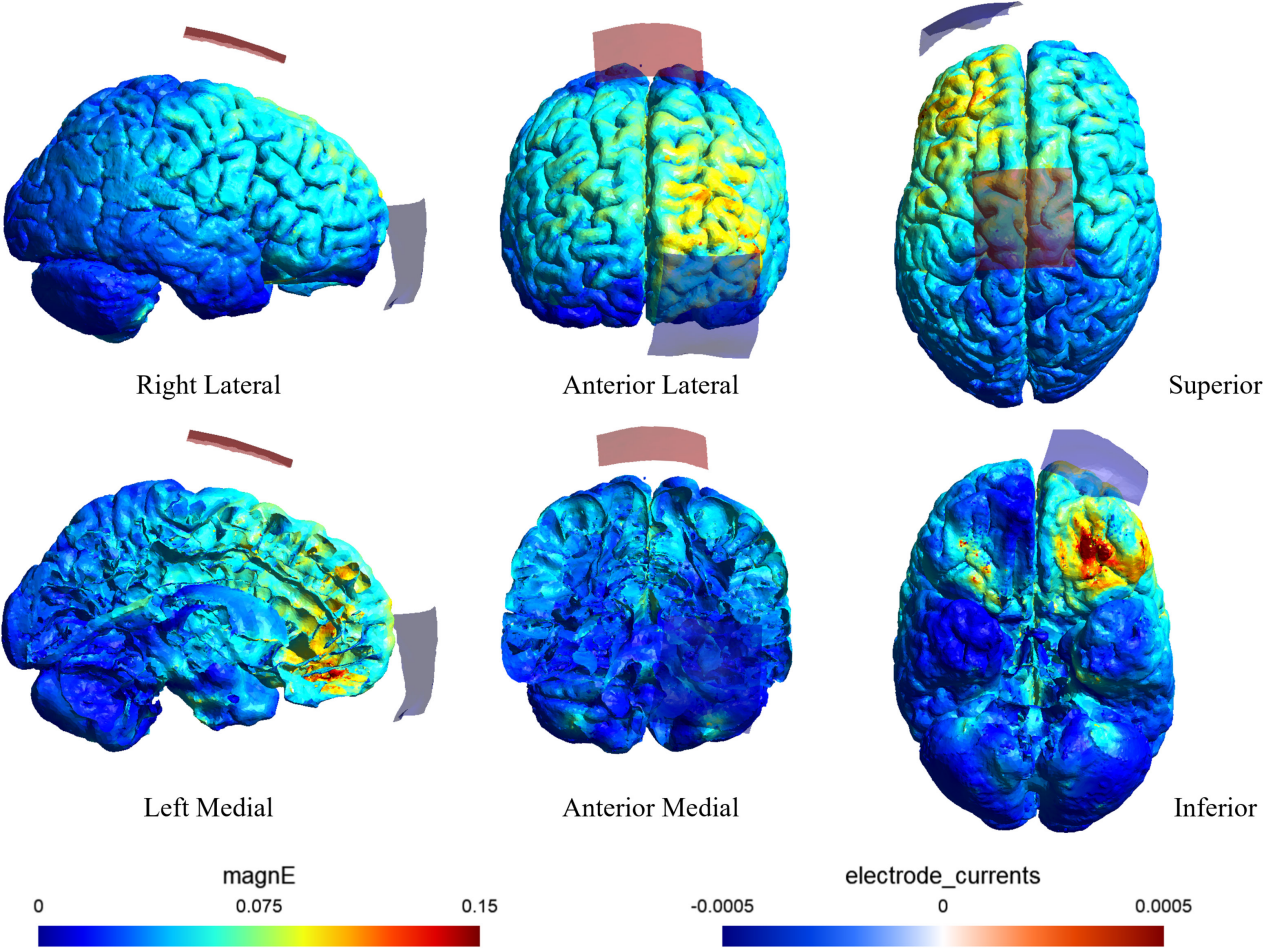
Electrode montage and electric field simulations for Cz‐tACS group. Electric field simulations using SimNIBS version 4.0.1, showing the electrode montage and simulated electric field distribution for the Cz‐tACS group.

tACS was delivered using a DC‐stimulator (Eldith, neuroConn GmbH, Ilmenau, Germany) via two saline‐soaked surface sponge electrodes (5 × 5 cm, 25 cm^2^). For the M1‐tACS and sham groups, two electrodes were placed over the right M1 and the left supraorbital ridge. To determine the optimal site for M1 electrode placement, we used a magnetic stimulator (Magstim, Whitland, UK) equipped with a figure‐of‐eight coil (diameter: 9.5 cm). The right M1 hotspot was identified by locating a position around C4 (International 10–20 system) that consistently elicited the largest motor‐evoked potentials in the left first dorsal interosseous muscle upon transcranial magnetic stimulation (TMS). This site was designated as the center for M1 electrode placement. In the Cz‐tACS group, which served as the active sham stimulation group, two electrodes were placed over Cz (International 10–20 system) and the left supraorbital ridge. The tACS parameters were as follows: 1.0 mA intensity (peak‐to‐peak), 0.75 Hz frequency in slow oscillation, 30‐min duration, and 5‐s fade‐in/fade‐out cycles. For the sham group, M1 stimulation was applied with 10 s of fade‐in/fade‐out at 0.75 Hz. The tACS application followed established safety guidelines (Antal et al. 2017). Electric field distributions were simulated using the Ernie standard brain dataset in SimNIBS version 4.0.1 to confirm localized electric fields in the right M1 region and left frontal region for the M1‐tACS and sham groups (Figure 2). In contrast, the Cz‐tACS group showed no electric field in the M1 region, with localization only in the left frontal region (Figure 3). The Cz‐tACS group was included to determine whether the effects observed in the M1‐tACS group were specific to the hand area of M1, rather than resulting from unintended electric field distribution in the frontal region.

### 2.4 Experimental Procedure

**FIGURE 4:**
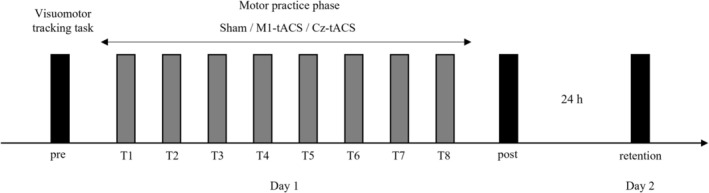
Schematic illustration of the experimental procedure.

The experimental procedure is illustrated in Figure 4. First, the maximum pinch force of the participants was measured to set the intensity of the visuomotor tracking task. After attaching tACS electrodes, participants performed a trial of the visuomotor tracking task before the motor practice (pre), followed by eight trials (T1–T8) with tACS, and one trial after the motor practice (post). A 2‐min break was provided between each trial to prevent participant fatigue. Additionally, one trial was performed 24 h later to assess the retention of motor skills (retention). Both Day 1 and Day 2 measurements were conducted between 12:00 and 18:00 h.

### 2.5 Statistical Analyses

In this study, we utilized power approximation curves to quantitatively assess the progression of motor learning. This modeling approach addressed the error rates in the visuomotor tracking task. The error rates decreased rapidly during the initial stages and declined gradually before approaching a plateau. The power function model is well‐suited to capturing such nonlinear learning dynamics in visuomotor skill acquisition (Cowley et al. 2019). Therefore, the power approximation index was employed as a motor learning indicator. For each participant, we derived power approximation curves describing the error rate trajectory before and after training and compared the resulting indices across groups (y = *a* x^*b*^, where y is the error rate, x is the number of trials, *a* is the proportional constant, and *b* is the power approximation index) (Prasertsakul et al. 2018). A smaller power approximation index indicates a more rapid decrease in error rate. Statistical analysis was conducted using SPSS Statistics version 25 (IBM). The Shapiro–Wilk test was used for normality testing. Based on the results, the Kruskal–Wallis test was used to compare power approximation indices and the mean coefficient of determination (*R* ^2^) of the power approximation curve between groups. The mean coefficient of determination was compared to ensure that the goodness of fit of the approximation curves did not differ between groups. The Kruskal–Wallis test was also used to compare error rates before motor practice, after motor practice, and on the following day in each group. The effect size was evaluated using *η* ^2^ (small effect size: 0.01, moderate effect size: 0.06, large effect size: 0.14). Post hoc comparisons were performed using the Dunn test using Bonferroni correction. The significance level was set at 5%.

## 3 Results

**FIGURE 5:**
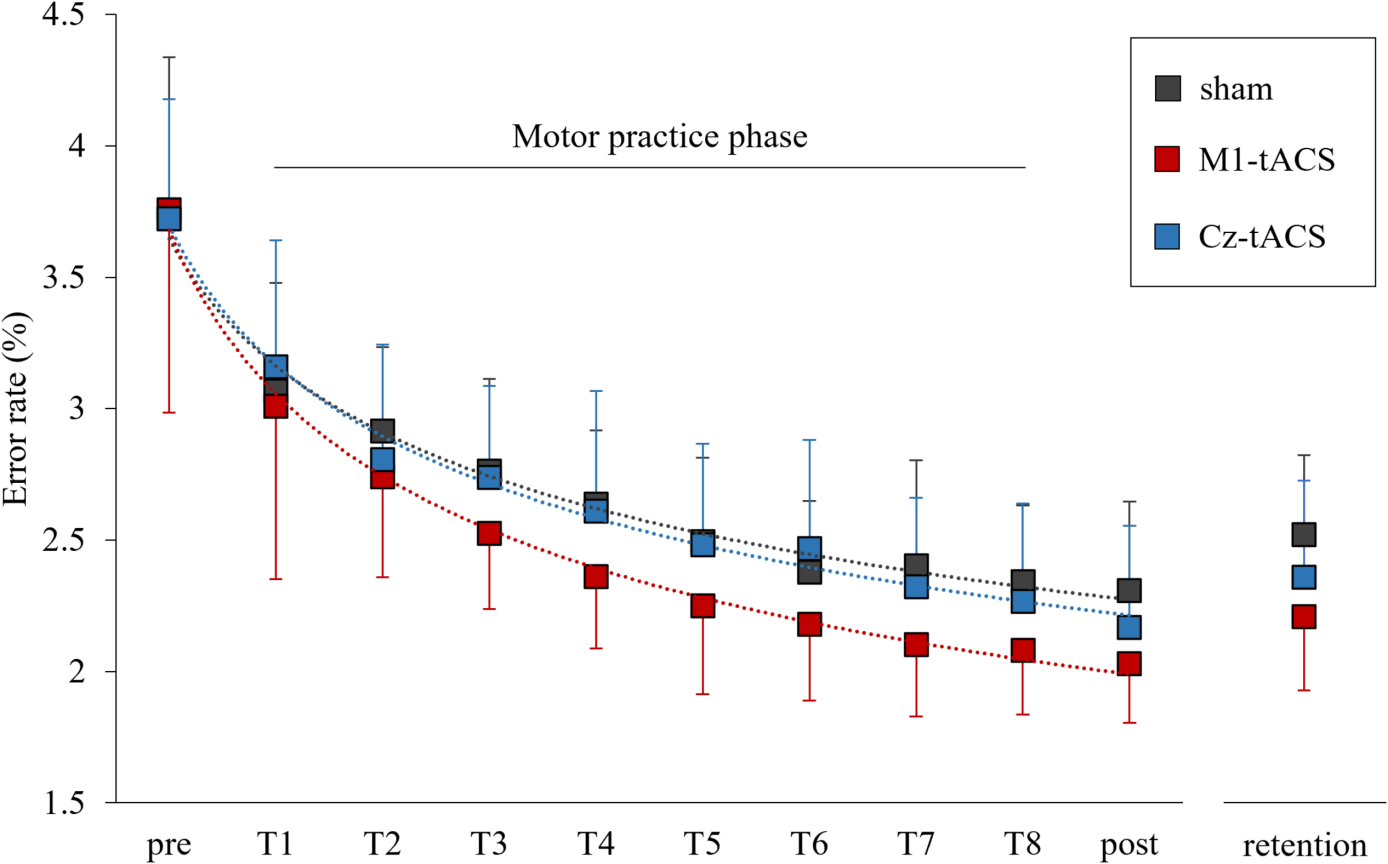
Change in error rates in each group. The dashed line shows the power approximation curve. Error bars indicate standard deviation.

**FIGURE 6:**
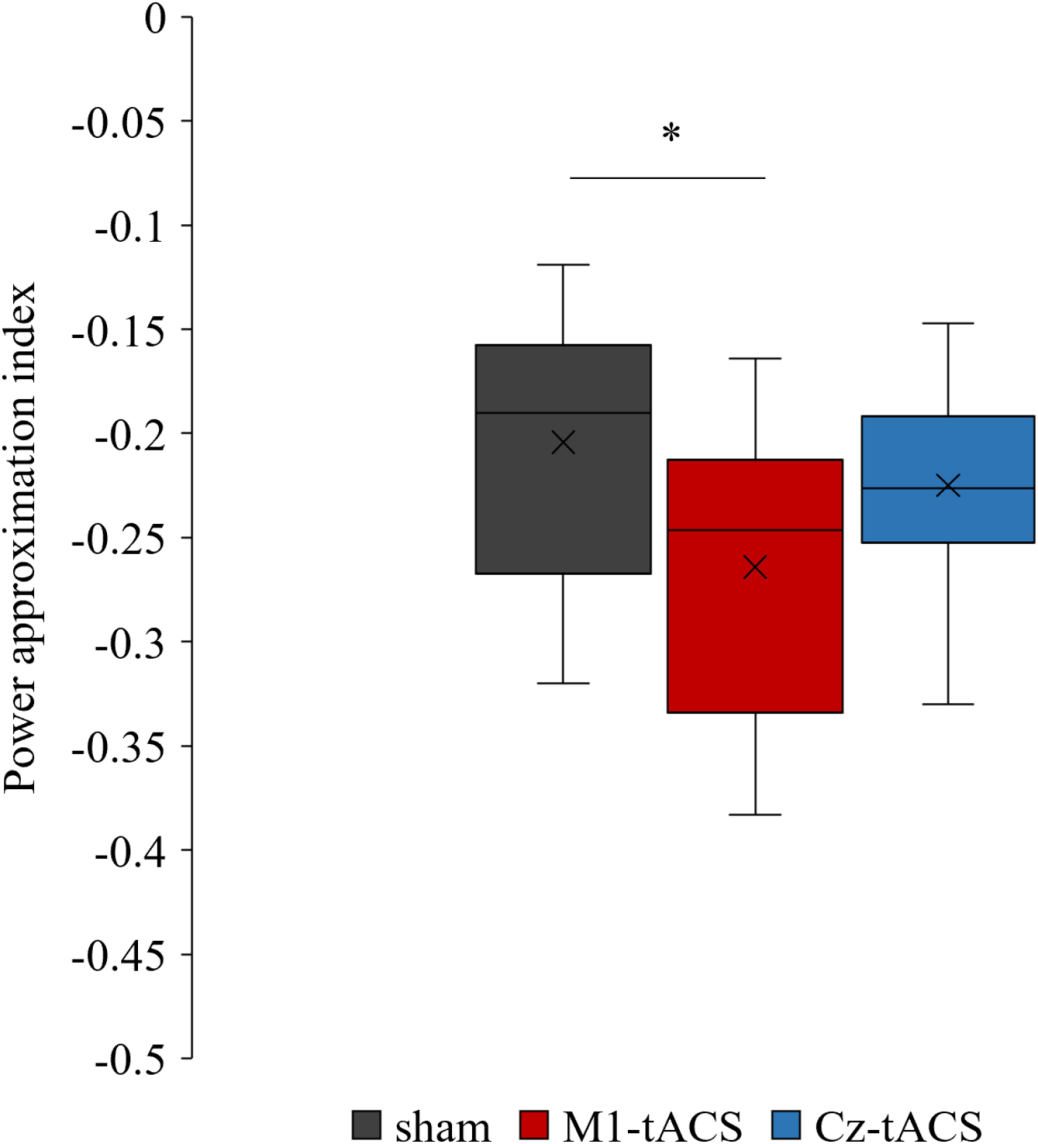
Power approximation index in each group. The cross mark indicates the average value. *p < 0.05 (Bonferroni correction).

All 54 subjects completed the assessments without interruption. Figure 5 shows the change in error rates for each group. The median coefficient of determination (*R* ^2^) of the power approximation curve was 0.851 [interquartile range (IQR): 0.719–0.933] for the sham group, 0.903 [IQR: 0.852–0.940] for the M1‐tACS group, and 0.872 [IQR: 0.819–0.925] for the Cz‐tACS group. The Kruskal–Wallis test indicated no significant differences in *R* ^2^ values among these groups [H(2) = 2.330, *p* = 0.312, *η* ^*2*^ = 0.006, a small effect]. Figure 6 shows the power approximation index in each group. The Kruskal–Wallis test revealed a significant groups difference in this index [H(2) = 6.553, *p* = 0.038, *η* ^*2*^ = 0.151, a large effect]. Post hoc pairwise comparisons showed that the power approximation index for the M1‐tACS group was significantly smaller compared with the sham group (corrected *p* = 0.033). There were no significant differences between the power approximation index of the sham and Cz‐tACS groups (corrected *p* = 0.927) or between the power approximation index of the M1‐tACS and Cz‐tACS groups (corrected *p* = 0.381).

**FIGURE 7:**
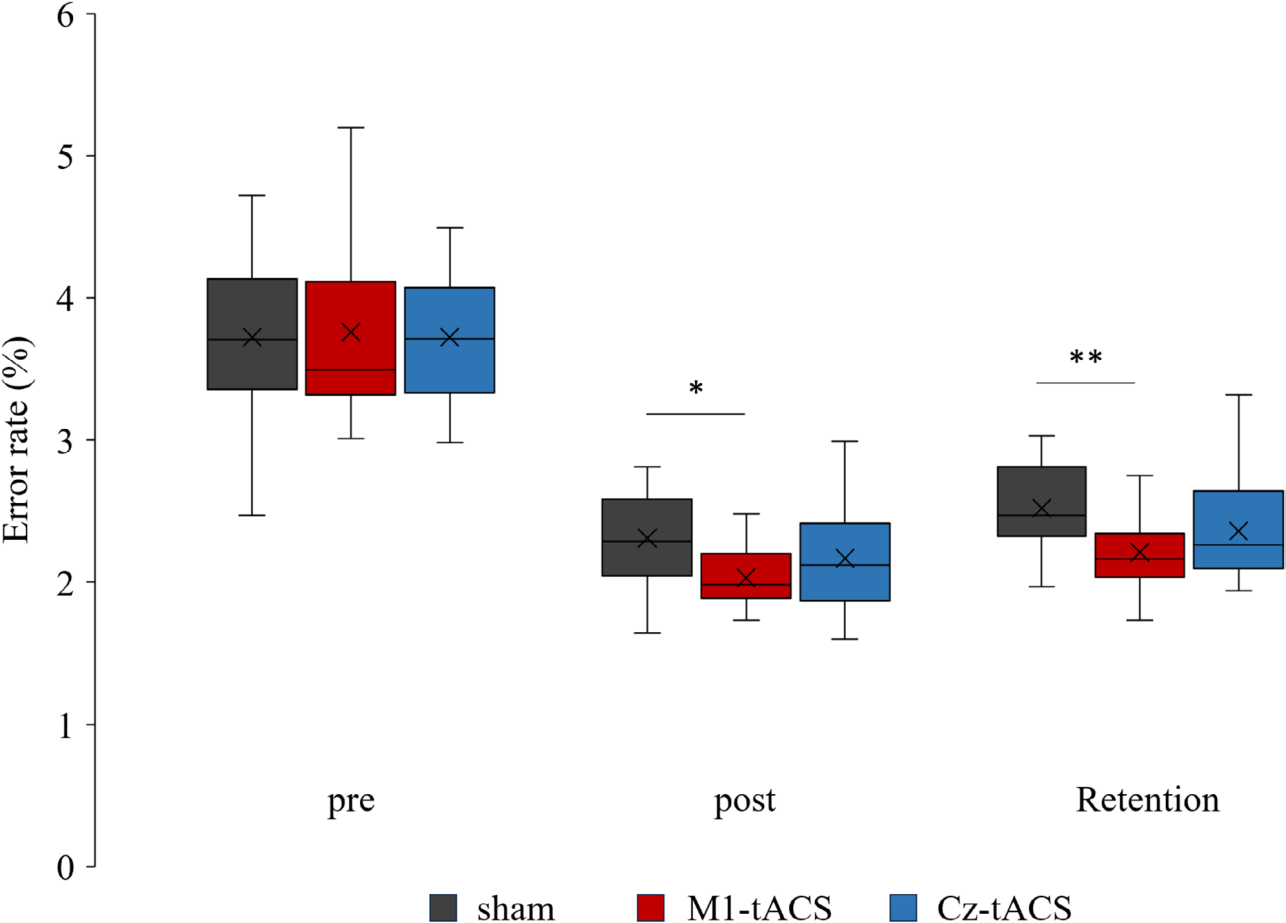
Error rates in each group. The cross mark indicates the average value. *p < 0.05, **p < 0.01 (Bonferroni correction).

Figure 7 displays error rates for pre, post, and retention trials for each group. The Kruskal–Wallis test indicated a significant group difference in posttrial error rates [H(2) = 6.220, *p* = 0.045, *η* ^*2*^ = 0.116, a large effect]. Based on post hoc pairwise comparisons, the M1‐tACS group had significantly lower error rates postpractice than the sham group (corrected *p* = 0.039). However, no significant differences were found between the sham and Cz‐tACS groups (corrected *p* = 0.462) or between the M1‐tACS and Cz‐tACS groups (corrected *p* = 0.868). Retention trial error rates also differed significantly among groups, as evidenced by the Kruskal–Wallis test [H(2) = 9.380, *p* = 0.009, *η* ^*2*^ = 0.143, a large effect], with post hoc pairwise comparisons demonstrating that the M1‐tACS group also showed a significantly lower error rate in the retention trial than the sham group (corrected *p* = 0.007). No significant differences were found between the sham and Cz‐tACS groups (corrected *p* = 0.174) or between the M1‐tACS and Cz‐tACS groups (corrected *p* = 0.771) in this respect. Prepractice error rates did not differ significantly among the groups [H(2) = 0.041, *p* = 0.980, *η* ^*2*^ < 0.001, a small effect].

## 4 Discussion

This study demonstrated that applying slow‐oscillatory tACS (0.75 Hz) to M1 during a visuomotor task enhanced motor skill acquisition, with improvements persisting into the following day. This enhancement was not observed in the sham or Cz‐tACS groups, indicating that the effect is specific to stimulation of the M1 hand area and not attributable to unintended electric fields from the frontal electrode. Although the absence of a difference between the M1‐tACS and Cz‐tACS groups warrants cautious interpretation, improvements confined to the M1‐tACS group modestly support the site‐specific contribution of M1 stimulation. These findings likely result from entrainment mechanisms and neuroplastic changes induced by tACS.

tACS is a noninvasive brain stimulation technique that modulates cortical oscillatory brain activity. Previous studies have shown that the stimulation effect, which synchronizes cortical oscillatory brain activity to the stimulation frequency, can persist beyond the stimulation period (Helfrich et al. 2014; Kasten et al. 2016). For example, applying 0.75‐Hz stimulation to the frontal cortex has been reported to increase slow‐wave oscillatory activity (Marshall et al. 2006; Kirov et al. 2009). In the present study, applying 0.75‐Hz tACS to M1 may have similarly increased slow‐wave oscillatory activity in this region. Slow oscillations are implicated in the control of movement direction and speed during motor performance (Bansal et al. 2011; Mollazadeh et al. 2011), and declines in δ‐band activity within the sensorimotor cortex have been linked to motor errors (Yordanova et al. 2024). Evidence from stroke model rats and human patients further suggests that increased low‐frequency oscillatory activity is associated with improved motor function (Ramanathan et al. 2018). Based on prior research, it is plausible that the application of 0.75‐Hz tACS in this study increased endogenous slow‐wave oscillatory brain activity, modulating neural activity related to motor control and error processing, thereby enhancing motor skill acquisition. Additionally, visuomotor tracking tasks engage a distributed network encompassing the prefrontal, premotor, and parietal cortices as well as the putamen and cerebellum, which together support visual processing, sensorimotor integration, and error correction (Vaillancourt et al. 2003, 2006). These regions are closely associated with M1 during visuomotor learning, suggesting that the observed effects of 0.75‐Hz tACS over M1 may reflect modulation of this broader visuomotor network. Moreover, prior work has shown that applying 0.75‐Hz tACS to the prefrontal cortex increases δ‐band and θ‐band activity (Kirov et al. 2009). A similar effect occurring with M1 stimulation cannot be ruled out. Given that θ‐band activity is implicated in visuomotor performance (Cruikshank et al. 2012; Tomassini et al. 2017), increased θ‐band activity could have contributed to the observed enhancement in motor skill acquisition. Future research should incorporate brain wave measurements to better understand the specific oscillatory brain activity underlying motor skill acquisition, providing more insights into the involved neural mechanisms.

Neuroplasticity may also have contributed to the improvement observed in this study. The 30‐min 0.75‐Hz tACS intervention could have increased corticospinal excitability, promoting motor learning. Supporting this notion, a previous study reported that 16 min of 0.75‐Hz tACS over M1 increased motor‐evoked potential amplitudes measured with TMS (Geffen et al. 2021), suggesting an excitatory effect on the corticospinal tract. However, the precise mechanisms remain unclear. Although the relationship between increased motor‐evoked potentials and motor learning is still debated, several studies indicate that heightened M1 excitability is associated with improved motor performance (Gallasch et al. 2009; Cirillo et al. 2011; Fujiyama et al. 2017; van de Ruit and Grey 2019). Taken together, these findings raise the possibility that the 30‐min intervention facilitated motor learning through increased M1 excitability.

Some limitations of this study should be acknowledged. First, we did not measure brain activity using electroencephalography (EEG) or TMS, which hindered elucidation of the underlying neurological mechanisms. Thus, our discussion of these mechanisms remains speculative. Future research should incorporate EEG to measure changes in slow‐wave (delta and theta) activity in M1 and TMS to assess corticospinal excitability. Second, we did not address frequency specificity, leaving it unclear whether the observed effects are due to frequency‐specific effects of slow‐wave stimulation. Subsequent studies should compare multiple slow‐wave frequencies and evaluate their effects relative to other tACS bands (e.g., alpha, beta, and gamma) to determine optimal stimulation parameters and delineate the roles of distinct oscillations. Third, no significant differences were observed between the M1‐tACS and Cz‐tACS groups. The lack of group‐level effects precludes attributing improvements solely to M1 stimulation. Nevertheless, because gains occurred only in the M1‐tACS group and were absent in the sham and Cz‐tACS groups, our findings support a potential role for M1‐targeted slow‐oscillatory stimulation. Despite these limitations, this study is the first to demonstrate that 0.75‐Hz tACS over M1 enhances motor skill acquisition, offering an important step toward understanding the contribution of slow‐oscillatory stimulation to motor learning.

## 5 Conclusion

This study demonstrated that applying 0.75‐Hz tACS to M1 enhanced visuomotor skill acquisition, likely through entrainment and neuroplastic mechanisms. In contrast, stimulation primarily targeting frontal regions did not yield similar benefits, suggesting that the facilitatory effects of 0.75‐Hz tACS are specific to M1 rather than to frontal stimulation.

## Author Contributions

S.M. contributed to the conceptualization, methodology, investigation, and formal analysis and was involved in writing the original draft as well as reviewing and editing the manuscript. Y.I. contributed to the conceptualization and methodology. K.I. was involved in the conceptualization, methodology, investigation, and formal analysis. S.Y. contributed to the methodology and investigation. N.O. performed the formal analysis. H.O. was involved in writing the original draft and in reviewing and editing the manuscript. All authors read and approved the final manuscript.

## Ethics Statement

The study was approved by the Ethics Committee of the Niigata University of Health and Welfare (approval number 19440‐241213), and the study was conducted in accordance with the principles of the Declaration of Helsinki. Written informed consent was obtained from all subjects.

## Consent

The authors have nothing to report.

## Conflicts of Interest

The authors declare no conflicts of interest.

## Peer Review

The peer review history for this article is available at https://www.webofscience.com/api/gateway/wos/peer‐review/10.1111/ejn.70269.

## Data Availability

The datasets used and/or analyzed during the current study are available from the corresponding author on reasonable request.

## References

[ejn70269-bib-0001] Antal, A. , I. Alekseichuk , M. Bikson , et al. 2017. “Low Intensity Transcranial Electric Stimulation: Safety, Ethical, Legal Regulatory and Application Guidelines.” Clinical Neurophysiology 128: 1774–1809. 10.1016/j.clinph.2017.06.001. 28709880 PMC5985830

[ejn70269-bib-0002] Antal, A. , and C. S. Herrmann . 2016. “Transcranial Alternating Current and Random Noise Stimulation: Possible Mechanisms.” Neural Plasticity 2016: 3616807. 10.1155/2016/3616807. 27242932 PMC4868897

[ejn70269-bib-0003] Bansal, A. K. , C. E. Vargas‐Irwin , W. Truccolo , and J. P. Donoghue . 2011. “Relationships Among Low‐Frequency Local Field Potentials, Spiking Activity, and Three‐Dimensional Reach and Grasp Kinematics in Primary Motor and Ventral Premotor Cortices.” Journal of Neurophysiology 105: 1603–1619. 10.1152/jn.00532.2010. 21273313 PMC3075284

[ejn70269-bib-0004] Bradley, C. , J. Elliott , S. Dudley , G. A. Kieseker , J. B. Mattingley , and M. V. Sale . 2022. “Slow‐Oscillatory tACS Does Not Modulate Human Motor Cortical Response to Repeated Plasticity Paradigms.” Experimental Brain Research 240: 2965–2979. 10.1007/s00221-022-06462-z. 36173425 PMC9587974

[ejn70269-bib-0005] Cirillo, J. , G. Todd , and J. G. Semmler . 2011. “Corticomotor Excitability and Plasticity Following Complex Visuomotor Training in Young and Old Adults.” European Journal of Neuroscience 34: 1847–1856. 10.1111/j.1460-9568.2011.07870.x. 22004476

[ejn70269-bib-0006] Cowley, B. U. , J. Palomäki , T. Tammi , et al. 2019. “Flow Experiences During Visuomotor Skill Acquisition Reflect Deviation From A Power‐Law Learning Curve, but Not Overall Level of Skill.” Frontiers in Psychology 10: 1126. 10.3389/fpsyg.2019.01126. 31156519 PMC6530424

[ejn70269-bib-0007] Cruikshank, L. C. , A. Singhal , M. Hueppelsheuser , and J. B. Caplan . 2012. “Theta Oscillations Reflect a Putative Neural Mechanism for Human Sensorimotor Integration.” Journal of Neurophysiology 107: 65–77. 10.1152/jn.00893.2010. 21975453

[ejn70269-bib-0008] Fujiyama, H. , M. R. Hinder , A. Barzideh , et al. 2017. “Preconditioning tDCS Facilitates Subsequent tDCS Effect on Skill Acquisition in Older Adults.” Neurobiology of Aging 51: 31–42. 10.1016/j.neurobiolaging.2016.11.012. 28033506

[ejn70269-bib-0009] Gallasch, E. , M. Christova , M. Krenn , A. Kossev , and D. Rafolt . 2009. “Changes in Motor Cortex Excitability Following Training of a Novel Goal‐Directed Motor Task.” European Journal of Applied Physiology 105: 47–54. 10.1007/s00421-008-0871-y. 18807065

[ejn70269-bib-0010] Geffen, A. , N. Bland , and M. V. Sale . 2021. “Effects of Slow Oscillatory Transcranial Alternating Current Stimulation on Motor Cortical Excitability Assessed by Transcranial Magnetic Stimulation.” Frontiers in Human Neuroscience 15: 726604. 10.3389/fnhum.2021.726604. 34588969 PMC8473706

[ejn70269-bib-0011] Giovanni, A. , F. Capone , L. Di Biase , et al. 2017. “Oscillatory Activities in Neurological Disorders of Elderly: Biomarkers to Target for Neuromodulation.” Frontiers in Aging Neuroscience 9: 189. 10.3389/fnagi.2017.00189. 28659788 PMC5468377

[ejn70269-bib-0012] Helfrich, R. F. , T. R. Schneider , S. Rach , S. A. Trautmann‐Lengsfeld , A. K. Engel , and C. S. Herrmann . 2014. “Entrainment of Brain Oscillations by Transcranial Alternating Current Stimulation.” Current Biology 24: 333–339. 10.1016/j.cub.2013.12.041. 24461998

[ejn70269-bib-0013] Kasten, F. H. , J. Dowsett , and C. S. Herrmann . 2016. “Sustained Aftereffect of α‐tACS Lasts Up to 70 Min After Stimulation.” Frontiers in Human Neuroscience 10: 245. 10.3389/fnhum.2016.00245. 27252642 PMC4879138

[ejn70269-bib-0014] King, B. R. , K. Hoedlmoser , F. Hirschauer , N. Dolfen , and G. Albouy . 2017. “Sleeping on the Motor Engram: The Multifaceted Nature of Sleep‐Related Motor Memory Consolidation.” Neuroscience and Biobehavioral Reviews 80: 1–22. 10.1016/j.neubiorev.2017.04.026. 28465166

[ejn70269-bib-0015] Kirov, R. , C. Weiss , H. R. Siebner , J. Born , and L. Marshall . 2009. “Slow Oscillation Electrical Brain Stimulation During Waking Promotes EEG Theta Activity and Memory Encoding.” Proceedings of the National Academy of Sciences of the United States of America 106: 15460–15465. 10.1073/pnas.0904438106. 19706399 PMC2730962

[ejn70269-bib-0016] Marshall, L. , H. Helgadóttir , M. Mölle , and J. Born . 2006. “Boosting Slow Oscillations During Sleep Potentiates Memory.” Nature 444: 610–613. 10.1038/nature05278. 17086200

[ejn70269-bib-0017] McNally, M. , G. Byczynski , and S. Vanneste . 2024. “An Overview of the Effects and Mechanisms of Transcranial Stimulation Frequency on Motor Learning.” Journal of Neuroengineering and Rehabilitation 21: 157. 10.1186/s12984-024-01464-0. 39267118 PMC11391832

[ejn70269-bib-0018] Mollazadeh, M. , V. Aggarwal , A. G. Davidson , A. J. Law , N. V. Thakor , and M. H. Schieber . 2011. “Spatiotemporal Variation of Multiple Neurophysiological Signals in the Primary Motor Cortex During Dexterous Reach‐to‐Grasp Movements.” Journal of Neuroscience 31: 15531–15543. 10.1523/JNEUROSCI.2999-11.2011. 22031899 PMC3246371

[ejn70269-bib-0019] Prasertsakul, T. , P. Kaimuk , W. Chinjenpradit , W. Limroongreungrat , and W. Charoensuk . 2018. “The Effect of Virtual Reality‐Based Balance Training on Motor Learning and Postural Control in Healthy Adults: A Randomized Preliminary Study.” Biomedical Engineering Online 17: 124. 10.1186/s12938-018-0550-0. 30227884 PMC6145375

[ejn70269-bib-0020] Ramanathan, D. S. , L. Guo , T. Gulati , et al. 2018. “Low‐Frequency Cortical Activity Is a Neuromodulatory Target That Tracks Recovery After Stroke.” Nature Medicine 24: 1257–1267. 10.1038/s41591-018-0058-y. PMC6093781 29915259

[ejn70269-bib-0021] Rostami, M. , A. Lee , A. K. Frazer , et al. 2023. “Determining the Corticospinal, Intracortical and Motor Function Responses to Transcranial Alternating Current Stimulation of the Motor Cortex in Healthy Adults: A Systematic Review and Meta‐Analysis.” Brain Research 1822: 148650. 10.1016/j.brainres.2023.148650. 39491217

[ejn70269-bib-0022] Sale, M. V. , and A. Kuzovina . 2022. “Motor Training is Improved by Concurrent Application of Slow Oscillating Transcranial Alternating Current Stimulation to Motor Cortex.” BMC Neuroscience 23: 45. 10.1186/s12868-022-00731-x. 35840886 PMC9287859

[ejn70269-bib-0023] Tomassini, A. , L. Ambrogioni , W. P. Medendorp , and E. Maris . 2017. “Theta Oscillations Locked to Intended Actions Rhythmically Modulate Perception.” eLife 6: e25618. 10.7554/eLife.25618. 28686161 PMC5553936

[ejn70269-bib-0024] Vaillancourt, D. E. , M. A. Mayka , and D. M. Corcos . 2006. “Intermittent Visuomotor Processing in the Human Cerebellum, Parietal Cortex, and Premotor Cortex.” Journal of Neurophysiology 95: 922–931. 10.1152/jn.00718.2005. 16267114 PMC2366036

[ejn70269-bib-0025] Vaillancourt, D. E. , K. R. Thulborn , and D. M. Corcos . 2003. “Neural Basis for the Processes That Underlie Visually Guided and Internally Guided Force Control in Humans.” Journal of Neurophysiology 90: 3330–3340. 10.1152/jn.00394.2003. 12840082

[ejn70269-bib-0026] van de Ruit, M. , and M. J. Grey . 2019. “Interindividual Variability in Use‐Dependent Plasticity Following Visuomotor Learning: The Effect of Handedness and Muscle Trained.” Journal of Motor Behavior 51: 171–184. 10.1080/00222895.2018.1446125. 29611783

[ejn70269-bib-0027] Yamamoto, S. , S. Miyaguchi , T. Ogawa , Y. Inukai , N. Otsuru , and H. Onishi . 2024. “Effects of Transcranial Alternating Current Stimulation to the Supplementary Motor Area on Motor Learning.” Frontiers in Behavioral Neuroscience 18: 1378059. 10.3389/fnbeh.2024.1378059. 38741685 PMC11089168

[ejn70269-bib-0028] Yordanova, J. , M. Falkenstein , and V. Kolev . 2024. “Motor Oscillations Reveal New Correlates of Error Processing in the Human Brain.” Scientific Reports 14: 5624. 10.1038/s41598-024-56223-x. 38454108 PMC10920772

